# Gene-Based Association Mapping for Dental Caries in The GENEVA Consortium

**Published:** 2020-04-15

**Authors:** Yueyao Wang, Dipankar Bandyopadhyay, John R. Shaffer, Xiaowei Wu

**Affiliations:** 1 Department of Statistics, Virginia Polytechnic Institute & State University, Blacksburg, VA; 2 Department of Biostatistics, Virginia Commonwealth University, Richmond, VA; 3 Department of Human Genetics, University of Pittsburgh, Pittsburgh, PA

**Keywords:** Adaptive-Weight Burden Test, Dental Caries, GWAS, GENEVA consortium

## Abstract

**Objective:**

Dental caries is a multifactorial disease with high prevalence in both children and adults. Recent genome-wide association studies (GWASs) have revealed that genetic factors play an important role in caries incidence. However, existing methods are not sufficient to identify caries-associated genes, due to the complex correlation structure of caries GWAS data, and lack of appropriate summarization at the gene level. This paper attempts to address that by analyzing data from the Gene, Environment Association Studies (GENEVA) consortium.

**Methods:**

We investigated gene-based genetic associations for dental caries based on genome-wide data derived from the GENEVA database, with adjustment to covariates, linkage disequilibrium among single-nucleotide polymorphisms, and family relations, in sampled individuals.

**Results:**

Several suggestive genes were identified, in which some of them have been previously found to have potential biological functions on cariogenesis.

**Conclusions:**

By comparing the gene sets identified from gene-based and SNP-based association testing methods, we found a non-negligible overlap, which indicates that our gene-based analysis can provide substantial supplement to the traditional GWAS analysis.

## Introduction

Dental caries is a chronic, transmissible disease caused by activities of bacteria [1]. Among the common disorders in humans, caries has the highest prevalence, affecting 2.43 billion people worldwide in their permanent teeth and 620 million children in their primary teeth [2]. It is known that multiple factors contribute to a person’s risk for caries development and progression. Besides environmental factors (diet, oral hygiene, etc) and host factors related to subjects’ oral conditions, genetic factors have been shown to play an important role in caries etiology [3–5].

Analyzing genome-wide data generated from epidemiological studies of dental health opens up cost-effective opportunities to uncover how genetic factors affect inherited risks for dental caries. Previous genome-wide association studies (GWASs) for dental caries have identified several genes that may be associated with caries heritability [6], including enamel formation genes such as AMBN, AMELX, ENAM [7–9], taste receptor genes such as TAS2R38, TAS1R2 [10,11], and genes related to immunity such as HLA [12,13] and saliva such as PRH1 [14]. Although considerable efforts have been made to capture GWAS signals for dental caries, they are often limited to marginal association between caries traits and each individual single nucleotide polymorphism (SNP), hence may have limited power at the gene level (especially when evaluating the genetic effect of low-frequency minor alleles). Moreover, due to genotyping differences in different GWASs, findings from such analyses may exhibit inconsistencies, which pose difficulties for biological interpretations. On the other hand, SNP-set-based or gene-based association analyses [15–19] are receiving increasing attention since genes are the functional unit of the human genome and remain highly consistent across diverse human populations. However, most of these gene-based methods are not sufficient to account for linkage disequilibrium (LD) among SNPs, i.e., the non-random association of alleles at different loci. In fact, since caries GWASs are often conducted on related individuals, e.g., family trios or pedigree samples, it is critical to model the complex genotypic correlations caused by both LD and familial relation in order to improve the power of association testing.

In this paper, we propose to use a novel gene-based association mapping strategy to identify caries-associated genes based on genome-wide data from the GENE enVironment Association studies (GENEVA) consortium. Several suggestive genes were identified, in which some (such as PTPRD) have been previously found in other single-SNP-based GWASs to play plausible biological roles for dental caries. An interesting finding was obtained by comparing the gene sets identified separately from gene-based and SNP-based tests. The non-negligible overlap between these two sets suggests that the two types of analyses are not independent of each other and therefore results from gene-based association analyses may contain important signals relevant to cariogenesis, complementing those from the traditional single-SNP-based GWASs.

## Materials and Methods

### Dental caries data from the GENEVA program

Initiated in 2006, the GENEVA program aims to enhance the benefit of collaborative work to further maximize knowledge obtainable through GWAS [20]. One participating study (dbGaP accession: phs000095.v3.p1) of this program collected dental caries data for 5,291 phenotyped and 4,020 genotyped individuals (including cohort of families and unrelated individuals). The major phenotype variable “Tot_D1MFT” describes the status of subjects’ teeth, which was derived by counting all teeth with caries (including white spots). Specifically, a tooth is recorded as a D if a carious lesion(s) or both carious lesion(s) and a restoration are present; M if a tooth has been extracted due to caries; and F if a permanent or temporary filling is present. In addition to the tooth-level caries index, another index at tooth surface level, “Tot_D1MFS”, is also generated in a similar fashion. Eight sociodemographic and environmental variables are collected as covariates, including sex, age at the time of dental exam, source of water (city/public, well and other), tooth brushing frequency, saliva flow rate (calculated as the volume of saliva divided by 3 minutes: in unit ml/min); home water source fluoride level (obtain from home tap water: in unit ppm), educational attainment (up to high school, some college, four-year degree or beyond), presence of oral *S. mutans* present (yes, no). The genotype data were collected for a total of 589,735 SNPs on an Illumina BeadChip, among which 289,629 (49.11%) are located in 26,449 entrez genes (according to the human gene Ensembl dataset GRCh37). Based on the cleaned genotype data, a total of 20,197 (76.36%) entrez genes were found to contain more than one SNP in the genotype data

As part of the quality-control procedure, the genotyped individuals were further filtered by excluding those who met either of the following two criteria:

1. Empirical self-kinship coefficient ${\tilde{\phi }}_{\text{ii}}>0.525$, i.e., empirical inbreeding coefficient ${\tilde{\text{h}}}_{\text{i}}\geq 0.05$, or
2. Completeness ≤ 96%, where completeness is defined as the proportion of SNPs for which a given individual had genotypes called. Under these criteria, a subset of 652 samples from the GENEVA dental caries data, which have complete data in both phenotypes and genotypes, were selected for the analysis.

Among these 652 participants, 571 are from 201 families (nuclear families or parent-offspring trios), and the rest are unrelated individuals. Quality control on SNPs was conducted based on the following conditions:

- Call rate ≥ 96%, and
- Minor allele frequency (MAF) ≥ 1%.

To perform the gene-based test, the entire genotype matrix (rows corresponding to individuals and columns corresponding to SNPs) was split according to the starting and ending positions of each gene.

Each partition of the genotype matrix was further cleaned in order to avoid the possible non-singular problem regarding the following aspects: (1) columns corresponding non-polymorphic SNPs were excluded, and (2) given limited sample size, only columns with unique SNP data were retained whereas duplicated ones were eliminated. These quality assurance steps resulted in 26,831 genes in consideration.

### Statistical approach

The adaptive-weight burden test (ABT) is a retrospective, mixed model test for genetic association of quantitative traits on genotype data with complex correlations. In the GENEVA dental caries study, a group of individuals with known pedigree information were sampled for phenotype (the DMFT index), covariates (sex, age, etc), and genotypes (multiple SNPs in pre-defined gene regions). Under the gene-based testing setup, we denote the phenotype vector by Y, covariate matrix by Z, and genotype matrix by G (for each gene, size n × m . Then, the ABT analysis is conducted on a retrospective paradigm G|(Y,Z), i.e., by treating genotypes as random response in regression. The main advantage of such a retrospective model lies in its ability to directly model genotypic correlations across both SNPs (caused by LD) and samples (due to familial relation) [21], as well as to maintain robustness to phenotype model misspecification [22].

It has been shown that the ABT statistic

$${\text{S}}_{\text{ABT}}=\frac{{\text{V}}^{\text{T}}\text{G}{\left(\hat{\text{D}}\text{R}\hat{\text{D}}\right)}^{-1}{\text{G}}^{\text{T}}\text{V}}{{\text{V}}^{\text{T}}\Phi \text{V}}$$

Where $\text{V}={\hat{\Sigma }}_{0}^{-1}\left(\text{Y}-\text{Z}{\hat{\beta }}_{0}\right)$ is the transformed phenotypic residual under the null hypothesis of no genetic association, and the phenotype covariance matrix $\sum_{0}=\sigma_{\text{e}}^{\text{2}}\text{I}+\sigma_{\text{a}}^{\text{2}}\Phi$. Here, Φ is the kinship matrix of the sampled individuals, and, $\sigma_{\text{e}}^{\text{2}}$, $\sigma_{\text{a}}^{\text{2}}$ stand for variance due to random measurement error and variance attributed to additive polygenic random effects, respectively. The covariance matrix of multiple SNPs in a pre-defined gene region is denoted as DRD, where R is the LD correlation matrix and $D=diag\left\{\sigma_{j}\right\},1\leq j\leq m$ is a diagonal matrix of the standard deviations of the SNPs in genotype G. ABT adopts adaptive weights to collapse multiple SNPs in each pre-defined gene region to improve power of association testing. Its alternative view is a kernel test with the generalized Madsen–Browning $\text{W}={\left({\text{V}}^{\text{T}}\Phi \text{V}\right)}^{-1/2}{\left(\hat{\text{D}}\text{R}\hat{\text{D}}\right)}^{-1/2}$. The null distribution of S_ABT_ can be explicitly derived as a mixture of $\chi_{1}^{2}$ random variables each obtained from the eigen value of the matrix, WG^T^PGW, where

$$\text{P}={\hat{\Sigma }}_{0}^{-1}-{\hat{\Sigma }}_{0}^{-1}\text{Z}{\left({\text{Z}}^{\text{T}}{\hat{\Sigma }}_{0}^{-1}\text{Z}\right)}^{-1}{\text{Z}}^{\text{T}}{\hat{\Sigma }}_{0}^{-1},$$

In order to justify the results from the gene-based association analysis, we compared the gene set identified by gene-based test with that by SNP-based test. For consistency reasons, we chose to use MASTOR as the SNP-based test. MASTOR is a mixed-model, retrospective score test for genetic association with quantitative traits in samples with related individuals [22]. When using such a single-SNP test, we consider a gene to be significantly associated with the DMFT trait if there exists at least one SNP in that gene region with p-value less than the Bonferroni corrected nominal α=0.05/(#SNP in the gene), after adjusting for non-genetic covariates. Since all other settings for this study, including the samples, covariates, phenotypes/genotypes, and the retrospective analytical strategy, keep unchanged from the gene-based analysis, the gene sets identified by ABT and by MASTOR should be comparable. A simple chi-square test for independence can then be conducted with a 2×2 contingency table formed with the significant/insignificant number of genes identified by these two methods.

## Results

The GENEVA dental caries data (dbGaP accession: phs000095. v3.p1) include 5,291 phenotyped and 4,020 genotyped individuals. In this study, we focus on a subset of 652 participants who have complete data in the following phenotypic characteristics: gender, age, education group, water source, presence/absence of S. mutans, home tap water fluoride level, saliva flow, brush frequency, and the Decay-Missing-Filled (DMF) index. These characteristics are summarized in Table 1.

We note that, the 652 participants include both children and adults. The age ranges from 7 to 61 years, and 406 out of the total 652 are older than 18 at the time of examination. The majority of these participants are white (648 white, two multi or bi-racial, and two with missing values), therefore race was not included as a covariate in our study.

We use the adaptive-weight burden test [15] to perform gene-based association testing for the DMFT index in the 652 participants, adjusting for the above eight non-genetic covariates and properly addressing various types of genotypic correlations caused by both LD and familial relation. This test first obtains the transformed phenotypic residual from the phenotype model under the null hypothesis, and then collapses the genotypes of multiple SNPs in each gene region by using data-adaptive weights to achieve a powerful retrospective, gene-based test (details are provided in Statistical approach).

In the step of null phenotype model identification, two variance-component parameters and 10 fixed-effect regression coefficients for the non-genetic covariates (two indicators for water source categories) are estimated by the maximum likelihood method. These estimates are shown in Table 2. In covariance estimation, we notice that the estimated value of $\sigma_{\text{a}}^{2}/\sigma^{2}$, i.e., the narrow-sense heritability, is about 0.44, which is comparable with traditional heritability estimates of DMFT (or DMFS) in the permanent dentition of other family-based dental caries GWASs [23–25]. In covariates effects estimation, we see that at nominal level 0.05, three covariates sex, age, and *S. mutans* are significantly associated with the DMFT trait with positive coefficient estimates, indicating that patients with male gender, younger age, and absence of *S. mutans* are more likely to have lower DMFT measurements. The brush frequency, while expected to be positively associated with dental caries, seems to have a “boundary” effect, which turns out to be significant at nominal level 0.1 but not at 0.05.

**Figure 1:**
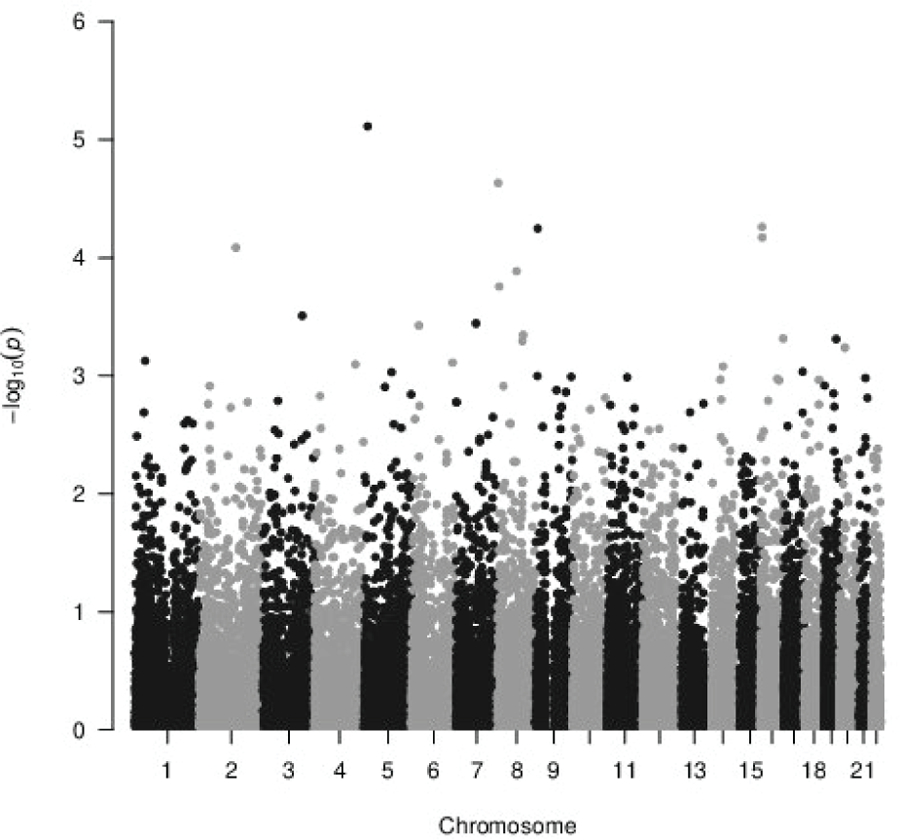
Manhattan Plot of Gene-based Association Testing P-values for GENEVA DMFT Data.

**Figure 2:**
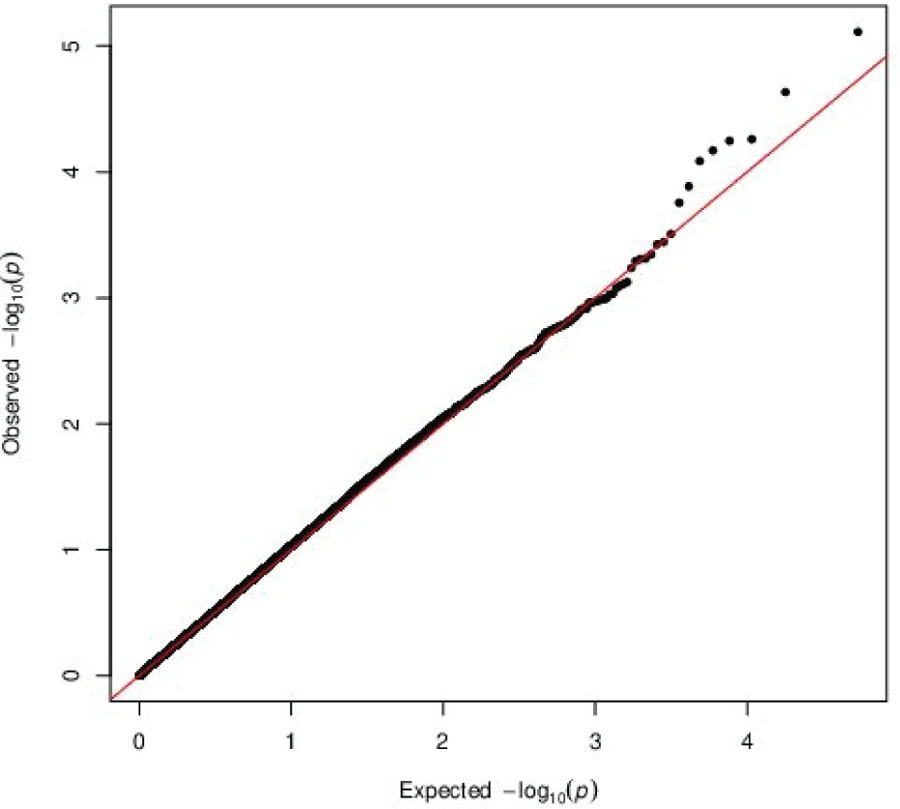
QQ Uniform Plot of Gene-based Association Testing P-values for GENEVA DMFT Data.

In this gene-based association analysis, the Manhattan plot and Quantile-Quantile (Q-Q) plot for a total number of 26,831 entrez genes on the human genome are shown in Figure 1 & 2, respectively. In Figure 2, the resulting genomic inflation factor λ is reported as 1.05 which is generally considered benign [26], suggesting that the gene-based p-values did not show substantial departure from the uniform distribution. Therefore, the extent of inflation due to population stratification or other confounders is negligible.

Table 3 lists the 10 top genes according to ranked ABT p-values for the GENEVA dental caries data. Among these 10 genes, PTPRD (MIM: 601598) has been recently reported to be associated with smooth and pit-and-fissure surface caries in the primary dentition in children by a single-SNP based GWAS (reported SNP: rs10958998, intronic, [27]). The PTPRD gene encodes a member of the PTP (protein tyrosine phosphatase) family which is known to be signaling molecules that regulate a variety of cellular processes including cell growth, differentiation, mitotic cycle, and oncogenic transformation.

Our study also identified five other genes that were reported in the GWAS catalogue [28] to be relevant to cariogenesis, namely FHIT (MIM: 601153), CNTN4 (MIM: 607280), CTNNA3 (MIM: 607667), IL17D (MIM: 607587), and CELF2 (MIM: 602538). The reported SNPs for these five genes are: rs9311745 (intron variant, [29]), rs17013524 (intron variant, [27]), rs2441755 (intron variant), rs735539 (intron variant), and rs11256676 (intergenic variant, [30]), respectively. The protein encoded by FHIT is a P1-P3-bis (5’-adenosyl) triphosphate hydrolase involved in purine metabolism. This gene encompasses the common fragile site FRA3B on chromosome 3, where carcinogen-induced damage can lead to translocations and aberrant transcripts. CNTN4 encodes a member of the contact in family of immunoglobulins. The encoded protein may play a role in the formation of axon connections in the developing nervous system. The CTNNA3 gene encodes a protein that belongs to the vinculin/alpha-catenin family and plays a role in cell-cell adhesion in muscle cells. IL17D encodes a cytokine that shares the sequence similarity with IL17. The treatment of endothelial cells with this cytokine has been shown to stimulate the production of other cytokines including IL6, IL8, and CSF2/GM-CSF.

The comparison of gene-based test and SNP-based test shows that, out of the total 26,831 genes, 1,053 exhibit significance by MASTOR, slightly less than the 1,468 genes found by ABT, and the intersection set, i.e., identified by both MASTOR and ABT, contains 688 genes. The contingency table is shown in Table 4, which gives a very significant p-value (< 2.2 × 10^−16^), indicating that the results from these two types of tests are not independent.

## Discussion

Traditional GWASs rely on screening the genome on the basis of SNPs. Though such a SNP-based association testing strategy has been shown successful in identifying susceptibility loci for several complex genetic diseases [31,32], challenges in GWASs still exist: First, SNP-based testing detects only marginal effects and may be underpowered to evaluate rare-variant effects due to their low allele frequencies. Second, since SNP-based testing only reports significant SNPs, identification of genes is usually ad hoc, depending on the relative location (intron, intergenic, noncoding, up/downstream, regulatory region, etc) of the identified SNPs to target genes, and interpretation of genetic effects at the gene level remains elusive. In contrast, gene-based testing assesses joint effects of multiple variants in a predefined gene region. Compared with SNP-based testing, gene-based testing has several appealing features. First, shifting the testing unit from SNP to gene generates more interpretable and replicable findings in gene function [33] and gene-gene interaction [34]. Second, by aggregating small signals from each SNP variant, gene-based testing usually achieves improved power, especially for low-frequency minor alleles [35]. Third, chance findings due to multiple testing will be reduced by using gene-wide instead of genome-wide significance level [10]. Finally, gene-based testing also lends itself to meta-analysis of combined data from multiple studies [36]. For these reasons, gene-based association testing is believed to be a natural approach for association analysis in the post-GWAS era of dense genotyping and fine mapping [37].

In this study, we performed gene-based association testing for dental caries data from the GENEVA consortium, with adjustment of phenotype covariates and accounting for LD in SNPs and familial relation in samples. We observed suggestive associations between the DMFT trait and several genes, some of which have been found to have plausible biological functions relevant to cariogenesis. Three non-genetic covariates sex, age, and *S. mutans* were found significantly associated with the DMFT trait, and the narrow-sense heritability was found comparable with traditional heritability estimates in previous family-based dental caries GWASs. Due to the differences between study designs and testing methods, it is not reasonable to compare the gene set identified by this study with that by other SNP-based GWASs. However, we attempted to compare the results from both gene- and SNP-based association testing on the basis of the GENEVA dental caries data. The comparison revealed that the gene-based test captured 65.34% genes that are significant in the SNP-based test. A further test for independence illustrated that, though gene-based association testing has a totally different mechanism, the identified genes are significantly overlapped with those by SNP-based association testing, given that two tests are performed on the same GENEVA data. Therefore, findings from this gene-based association testing may contain important signals relevant to cariogenesis and could complement those from the traditional SNP-based GWASs.

## Figures and Tables

**Table 1:** Sample Characteristics in the GENEVA Data.

Characteristics	
Sample size	652
Gender: male/female	281/371
Age at examination (mean ± sd)	25.4 ± 13.3
Education group^1^	452/115/85
Water source: city, public/well/other	492/147/13
Presenting *S. mutans* (%)	74.1
Home tap water fluoride level (mean ± sd)	0.7 ± 0.4
Saliva flow (mean ± sd)	0.2 ± 0.4
Brush more than once a day (%)	72.39
DMFT^2^ (mean ± sd)	8.5 ± 6.3
DMFS^3^ (mean ± sd)	17.6 ± 19.3

1. Education group has 3 ordinal categories: (i) up to high school; (ii) some college; and (iii) four-year degree or beyond.

2. DMFT: derived by counting all teeth (including both primary and permanent) that is decayed, missing, and filled.

3. DMFS: derived by counting all surfaces (including both primary and permanent) those are decayed, missing, and filled.

**Table 2:** Estimated Covariance and Covariate Effects in the GENEVA Data.

Parameters	Estimate	
Covariance Components		
$\sigma^{2*}$	33.211	
$\sigma_{a}^{2}/\sigma^{2}$	0.4437	
$\sigma_{e}^{2}/\sigma^{2}$	0.5563	
**Covariate Effects**	**Estimate (SE)**	**p-value**
Sex: Female	0.930 (0.468)	0.0471
Age	0.225 (0.020)	< 2 × 10^−16^
Water source: Well	0.185 (0.620)	0.766
Water source: Other	1.669 (1.682)	0.321
Brush frequency^†^	0.674 (0.348)	0.053
Saliva flow	−0.180 (0.582)	0.758
Home water fluoride level	0.714 (0.610)	0.243
Education level§	−0.612 (0.374)	0.103
*S. mutans*	2.134 (0.523)	5.13 × 10^−5^
Intercept	−1.365 (1.546)	0.378

**Note:** $*\sigma^{2}=\sigma_{a}^{2}+\sigma_{e}^{2}$

† : Brush frequency coded as: 1= more than 3 times per day, 2 = 3 times per day, 3 = 2 times per day, 4 = once a day, 5 = less than once a day. In the final samples, category 1 has zero observations, so category 2 is treated as the reference group.

§ : Education level is coded as: 1 = up to high school, 2 = some college, 3 = four-year degree or beyond.

**Table 3:** Strongest Association Signals in the GENEVA Data. DMFT-associated genes, with top 10 ABT ranked p-values are reported. Underlined genes have been previously identified to be associated with dental caries. MIM numbers of genes not mentioned in the text: CSMD1 (MIM: 608397), RBFOX1 (MIM: 605104), and CLRN1 (MIM: 606397).

Chr	Ensembl Gene	HGNC symbol	#SNP	p-value
5	ENSG00000248131	CT49	20	7.717 × 10^−6^
8	ENSG00000183117	CSMD1	1659	2.328 × 10^−5^
16	ENSG00000260411		713	5.491 × 10^−5^
9	ENSG00000153707	PTPRD	933	5.671 × 10^−5^
16	ENSG00000078328	RBFOX1	897	6.749 × 10^−5^
2	ENSG00000230569		1	8.217 × 10^−5^
8	ENSG00000253317		3	1.303 × 10^−4^
8	ENSG00000215372	ZNF705G	1	1.755 × 10^−4^
3	ENSG00000163646	CLRN1	18	3.106 × 10^−4^
7	ENSG00000214439	FAM185BP	14	3.608 × 10^−4^

**Table 4:** Comparison of Gene Sets Identified by Gene- and SNP-based Tests

SNP-based analysis (MASTOR)		
Gene-based analysis (ABT)	Significant	Non-significant
Significant	688	780
Non-significant	365	24,998
